# Harmful Algal Bloom Toxicity in *Lithobates catesbeiana* Tadpoles

**DOI:** 10.3390/toxins12060378

**Published:** 2020-06-08

**Authors:** Robin C. Su, Casey M. Meyers, Emily A. Warner, Jessica A. Garcia, Jeanine M. Refsnider, Apurva Lad, Joshua D. Breidenbach, Nikolai Modyanov, Deepak Malhotra, Steven T. Haller, David J. Kennedy

**Affiliations:** 1 Department of Medicine, The University of Toledo College of Medicine and Life Sciences, 3000 Arlington Avenue, Toledo, OH 43614, USA; rsu@rockets.utoledo.edu (R.C.S.); Emily.Warner2@rockets.utoledo.edu (E.A.W.); Apurva.Lad@rockets.utoledo.edu (A.L.); Joshua.Breidenbach@rockets.utoledo.edu (J.D.B.); Deepak.Malhotra@utoledo.edu (D.M.); 2 Department of Biology, Wittenberg University, Springfield, OH 45504, USA; meyersc@wittenberg.edu; 3 Department of Environmental Sciences, The University of Toledo, Toledo, OH 43606, USA; jgarcia12057@gmail.com (J.A.G.); jeanine.refsnider@utoledo.edu (J.M.R.); 4 Department of Physiology and Pharmacology, The University of Toledo College of Medicine and Life Sciences, Toledo, OH 43614, USA; Nikolai.Modyanov@utoledo.edu; 5 Department of Medical Microbiology and Immunology, The University of Toledo College of Medicine and Life Sciences, Toledo, OH 43614, USA

**Keywords:** harmful algal bloom, microcystins, tadpoles, intestines, liver, toxicity

## Abstract

Harmful algal blooms (HAB) have become a major health concern worldwide, not just to humans that consume and recreate on contaminated waters, but also to the fauna that inhabit the environments surrounding affected areas. HABs contain heterotrophic bacteria, cyanobacterial lipopolysaccharide, and cyanobacterial toxins such as microcystins, that can cause severe toxicity in many aquatic species as well as bioaccumulation within various organs. Thus, the possibility of trophic transference of this toxin through the food chain has potentially important health implications for other organisms in the related food web. While some species have developed adaptions to attenuate the toxic effects of HAB toxins, there are still numerous species that remain vulnerable, including *Lithobates catesbeiana* (American bullfrog) tadpoles. In the current study we demonstrate that acute, short-term exposure of tadpoles to HAB toxins containing 1 µg/L (1 nmol/L) of total microcystins for only 7 days results in significant liver and intestinal toxicity within tadpoles. Exposed tadpoles had increased intestinal diameter, decreased intestinal fold heights, and a constant number of intestinal folds, indicating pathological intestinal distension, similar to what is seen in various disease processes, such as toxic megacolon. HAB-toxin-exposed tadpoles also demonstrated hepatocyte hypertrophy with increased hepatocyte binucleation consistent with carcinogenic and oxidative processes within the liver. Both livers and intestines of HAB-toxin-exposed tadpoles demonstrated significant increases in protein carbonylation consistent with oxidative stress and damage. These findings demonstrate that short-term exposure to HAB toxins, including microcystins, can have significant adverse effects in amphibian populations. This acute, short-term toxicity highlights the need to evaluate the influence HAB toxins may have on other vulnerable species within the food web and how those may ultimately also impact human health.

## 1. Introduction

Cyanobacteria are a group of photosynthetic prokaryotes that exist in a wide range of environments and temperatures. Several species of these cyanobacteria produce a variety of secondary metabolites that can adversely affect the surrounding flora and fauna. These secondary metabolites are called cyanotoxins and are classified based on their chemical structure (cyclic peptides, alkaloids, and lipopolysaccharides) and toxicity based on their target organ system (hepatotoxins, neurotoxins, cytotoxins, and dermatotoxins). Amongst all the cyanotoxins, microcystins are produced by several strains of cyanobacteria [1].

Microcystin is produced by cyanobacteria, or blue-green algae, that contaminate freshwater environments around the world during harmful algal blooms (HABs) [2]. These HABs have alarmingly increased in frequency and severity annually [3]. HABs contain heterotrophic bacteria, cyanobacterial lipopolysaccharide, and cyanobacterial toxins, such as microcystins, that can cause severe toxicity in many aquatic species. In addition, microcystins have been known to have significant bioaccumulation within various organ systems of several species, and subsequently cause severe toxicity [3,4,5,6]. Recent studies, including several of our investigations, have used a combination of Solid Phase Extraction and Liquid Chromatography–Mass Spectrometry (LC–MS) methods to detect various congeners of microcystins in plasma, urine, and liver tissues of mice exposed to chronic, low doses of MC-LR [7,8,9] and others have reviewed MC-LR’s accumulation within animals and plants [1,10].

The effects of HAB toxins, such as microcystins, have been extensively studied in humans and have been recently reviewed [11]. In humans, microcystin can cause severe liver toxicity [5] and has even been categorized as a carcinogen by the International Agency for Research on Cancer [12,13]. Several cases of human exposure have been reported. One of the most notable events occurred in 1996, where 100 of 130 patients receiving dialysis at a clinic in Brazil experienced acute liver failure and 52 of those patients subsequently died due to a syndrome called “Caruaru Syndrome” [14,15,16]. It was later identified that the clinic was receiving untreated water and that the major driving factor leading to the documented deaths was intravenous exposure to microcystins [14,15,16].

The effects of microcystins are not limited to humans but are a major, direct health threat to the aquatic species inhabiting contaminated waters and the surrounding fauna. HAB toxins such as microcystins have been found to have bioaccumulation within fish, frogs, mussels, water snails, and various other aquatic species [17]. Extensively studied in fish, microcystins have been found to have a wide range of toxic effects, including abnormalities in embryo formation, growth rate retardation, and histopathology of liver, intestines, kidneys, heart, spleen, and gills [18]. Other species have shown microcystins to disrupt endocrine functions, damage the nervous system, cause hemorrhaging in the liver, and decrease folding in the stomach and intestines, resulting in decreased absorption of nutrients needed for proper growth and development [19,20].

HAB toxins such as microcystins also have detrimental effects on the reproductive system of many species and subsequently affect species survival. Microcystins can affect the quality of egg and sperm production and can cause developmental neuropathology within offspring [21]. Within new offspring, microcystins can also increase susceptibility to parasitism, cause growth malformations, cause cardiac defects, delay growth, and increase mortality [22]. Microcystins’ effects on zebrafish embryonic and larval mortality have been so pronounced that a 50% decrease in population density is anticipated over the next 100 years, increasing the threat of extinction in these populations [23].

While some species are more prone to toxic effects of HAB toxins such as microcystins, other species have developed mechanisms of HAB toxin resistance, such as crayfish [24]. Some species have developed higher tolerances to microcystins, such as grass carp [25]. Other species have even developed specific defense mechanisms, such as changing water depth to avoid higher concentrations of microcystins, detoxification of microcystins through glutathione and antioxidant enzymes, rapid excretion of microcystins, and the use of chemicals such as quercetin for preventing microcystin immunotoxicity [26]. However, even when resistant to microcystin’s toxic effects, crayfish still experience significant bioaccumulation of microcystins in their intestines and hepatopancreas [24]. Such accumulation in crayfish and other smaller species of aquatic wildlife, such as snails, poses a toxic risk for larger predators higher on the food chain [24,27]. This chain reaction creates potential trophic transference of microcystins from the smallest aquatic species all the way to humans.

To date, most studies of HAB toxicity in aquatic wildlife have been done in fish, mollusks, and shellfish. One of the populations that remain to be thoroughly evaluated for HAB effects is amphibians. In order to address this, we have characterized the acute, short-term effects of HAB toxins, including microcystins (i.e., total microcystins, which include intracellular and extracellular levels of all microcystin congeners), in the intestines and livers of *Lithobates catesbeiana* (American bullfrog) tadpoles. These tadpoles represent one of the populations that are frequently found to inhabit freshwater environments contaminated by HAB toxins.

## 2. Results

### 2.1. Intestinal Diameters

**Figure 1:**
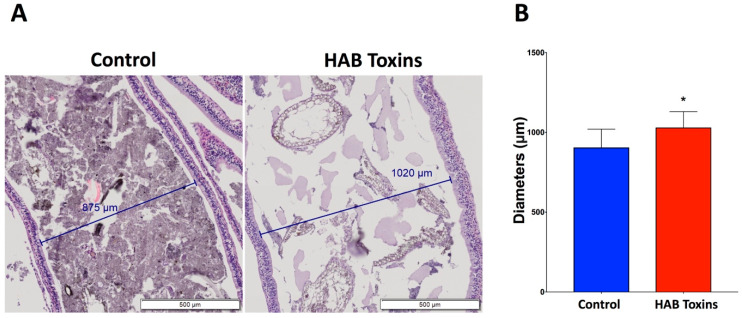
Tadpole intestinal diameters. (**A**) Hematoxylin and eosin (H&E)-stained intestinal sections reveal visibly larger intestinal diameters as well as decreased fecal content density in the harmful algal bloom (HAB)-toxin-exposed tadpoles as compared with the control tadpoles. (**B**) Quantitative analysis reveals significantly greater intestinal diameters in the HAB-toxin-exposed tadpoles as compared with the control tadpoles. Data presented indicate the mean ± SD (n = 10 tadpoles per group; 10 measurements taken per tadpole). * *p <* 0.05 by unpaired t-test vs. control group.

Histopathological analysis of hematoxylin and eosin (H&E)-stained intestinal sections from tadpoles revealed visibly distended intestines in HAB-toxin-exposed tadpoles as compared with control tadpoles that were exposed to normal pond water (Figure 1A). Further quantitative analysis confirmed that the intestinal diameters of HAB-toxin-exposed tadpoles were significantly greater than the intestinal diameters of control tadpoles (Figure 1B). We also noted a 64% decrease in the fecal content density in the HAB-toxin-exposed tadpoles as compared with the controls (41% fecal content density for HAB-toxin-exposed tadpoles vs. 15% fecal content density for control tadpoles, *p* < 0.0001).

### 2.2. Intestinal Fold Heights

**Figure 2:**
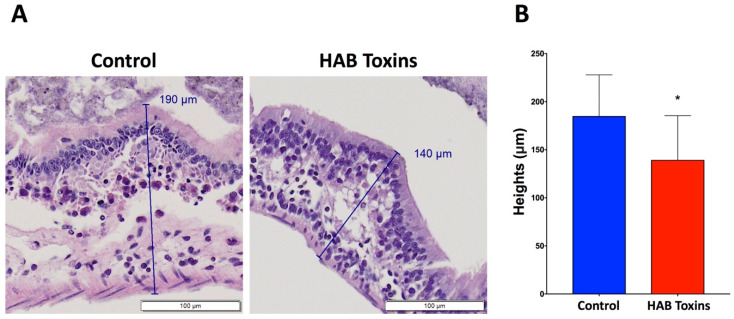
Tadpole intestinal fold heights. (**A**) H&E-stained intestinal sections reveal visibly shorter intestinal fold heights in the HAB-toxin-exposed tadpoles as compared with the control tadpoles. (**B**) Quantitative analysis reveals significantly shorter intestinal fold heights in the HAB-toxin-exposed tadpoles as compared with the control tadpoles. Data presented indicate the mean ± SD (n = 10 tadpoles per group; 20 measurements taken per tadpole). * *p <* 0.05 by unpaired t-test vs. control group.

Histopathological analysis of H&E-stained intestinal sections from tadpoles also revealed that intestinal folds were visibly shorter in height in the HAB-toxin-exposed tadpoles as compared with control tadpoles that were exposed to normal pond water (Figure 2A). Further quantitative analysis confirmed that the intestinal folds were significantly shorter in height in the HAB-toxin-exposed tadpoles as compared with control tadpoles (Figure 2B).

### 2.3. Intestinal Fold Numbers

**Figure 3:**
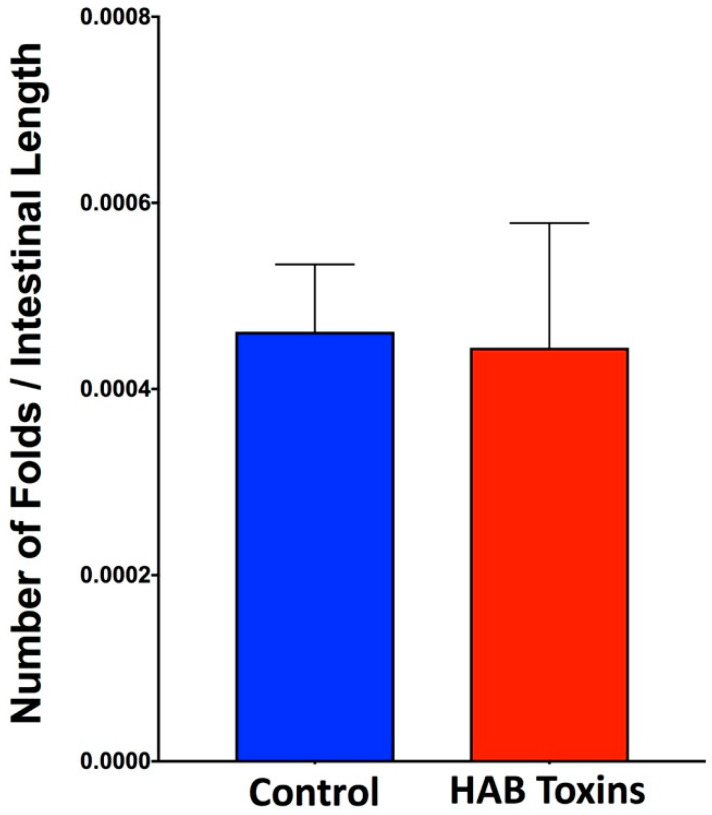
Normalized tadpole intestinal fold number. Total number of intestinal folds per tadpole was normalized to total intestinal length per tadpole. Data presented indicate the mean ± SD (n = 10 tadpoles per group).

The total number of intestinal folds per tadpole was normalized to the total length of intestine per tadpole. Normalized intestinal fold numbers revealed no significant differences between HAB-toxin-exposed tadpoles and control tadpoles exposed to normal pond water (Figure 3).

### 2.4. Hepatocyte Size

**Figure 4:**
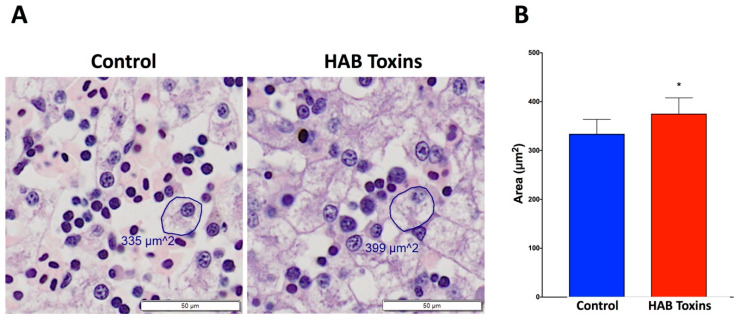
Hepatocyte sizes of tadpole liver sections. (**A**) H&E-stained liver sections reveal visibly larger hepatocytes in the HAB-toxin-exposed tadpoles as compared with the control tadpoles. (**B**) Quantitative analysis reveals significantly larger hepatocytes, as measured by surface area, in the HAB-toxin-exposed tadpoles as compared with the control tadpoles. Data presented indicate the mean ± SD (n = 10 tadpoles per group; 50 hepatocytes measured per tadpole). * *p <* 0.05 by unpaired *t*-test vs. control group.

Histopathological analysis of H&E-stained liver sections from tadpoles revealed visibly larger hepatocytes in HAB-toxin-exposed tadpoles as compared with control tadpoles that were exposed to normal pond water (Figure 4A). Further quantitative analysis confirmed that the hepatocyte sizes, as measured by surface area, of HAB-toxin-exposed tadpoles were significantly larger than the hepatocyte sizes of control tadpoles (Figure 4B).

### 2.5. Hepatocyte Binucleation

**Figure 5:**
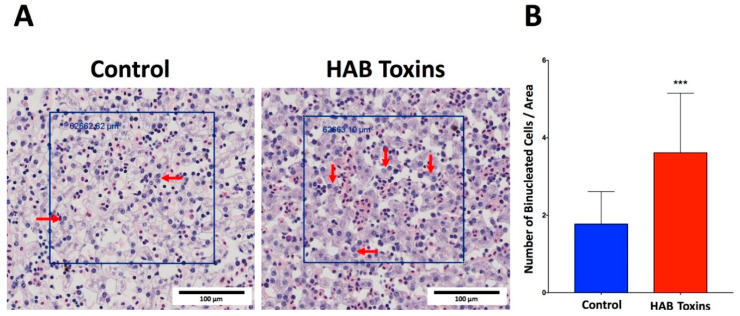
Hepatocyte binucleation. (**A**) H&E-stained liver sections reveal a visibly greater number of binucleated hepatocytes in the HAB-toxin-exposed tadpoles as compared with the control tadpoles. (**B**) Quantitative analysis reveals a significantly greater number of binucleated hepatocytes in the HAB-toxin-exposed tadpoles as compared with the control tadpoles. Data presented indicate the mean ± SD (n = 10 tadpoles per group; 5 areas measured from each quadrant the liver of each tadpole). *** *p <* 0.001 by Mann–Whitney rank sum test vs. control group.

Histopathological analysis of H&E-stained liver sections from tadpoles revealed a visibly greater number of binucleated hepatocytes in HAB-toxin-exposed tadpoles as compared with control tadpoles that were exposed to normal pond water (Figure 5A). Further quantitative analysis confirmed a significantly greater number of binucleated hepatocytes in HAB-toxin-exposed tadpole livers as compared with control tadpole livers (Figure 5B).

### 2.6. Immunohistochemistry for Protein Carbonylation

**Figure 6:**
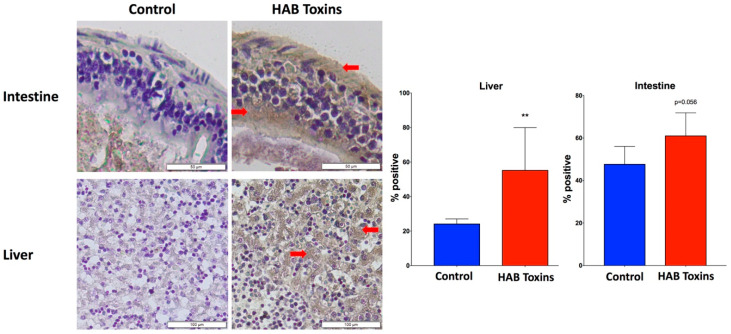
Immunohistochemical staining for protein carbonylation. Positive staining for protein carbonylation is demonstrated by diffuse brown 3,3′-Diaminobenzidine (DAB) staining within the intestinal wall and liver tissue as pointed out by red arrows. Immunohistochemical (IHC) staining for protein carbonylation revealed greater staining in intestine and liver tissues of HAB-toxin-exposed tadpoles as compared with control tadpoles. Data presented indicate the mean ± SD (n = 5 tadpoles per group). ** *p <* 0.01 by Mann–Whitney rank sum test vs. control group.

Intestine and liver tissue sections were processed for immunohistochemical (IHC) analysis of protein carbonylation as a marker of oxidative stress. Positive staining for protein carbonylation is demonstrated by diffuse brown 3,3′-Diaminobenzidine (DAB) staining within the intestinal wall and liver tissue as pointed out by red arrows (Figure 6). Analysis revealed visibly greater staining of intestine and liver tissue from HAB-toxin-exposed tadpoles as compared with control tadpoles that were exposed to normal pond water, signifying greater oxidative damage with HAB toxin exposure (Figure 6).

## 3. Discussion

HAB toxins have become a growing global health concern, not only for humans but also for the fauna that inhabit environments surrounding contaminated water sources. We aimed to investigate the acute effects of short-term exposure to HAB toxins containing microcystins on tadpoles, knowing that most previous studies have observed deleterious effects with long-term exposure.

In the current study, we have shown that just 7 days of HAB toxin exposure causes damaging effects in tadpole livers and intestines. Our results show that the intestines in HAB-toxin-exposed tadpoles were significantly more distended than in control tadpoles. The number of intestinal folds is similar between exposed and non-exposed tadpoles, and the actual intestinal fold heights are significantly shorter in HAB-toxin-exposed tadpoles compared with control tadpoles. Together, these suggest that there is intestinal distention in HAB-toxin-exposed tadpoles, with intestinal diameter expansion and flattening of intestinal fold heights.

Changes in intestinal diameter, fold height, and fold number have been previously shown to indicate intestinal remodeling in thyroid hormone receptor knock-out tadpoles [28]. Intestinal distension can be a result of a wide range of pathological processes. Bloating and distension are frequently seen with inflammatory diseases of the intestines, such as inflammatory bowel disease, and are seen in 96% of patients with irritable bowel syndrome [29]. Distension can also be a result of various infections [30]. A serious and life-threatening complication of inflammatory bowel processes and infections is toxic megacolon [31]. Toxic megacolon results from inflammation of the intestinal smooth muscle, paralysis, and subsequent distension [31]. Toxic megacolon is a serious health condition with a 13% mortality rate in humans [32]. We also noted that there is a 64% decrease in fecal content density within the intestinal lumens of HAB-toxin-exposed tadpoles as compared with control tadpoles. This finding is directly supportive of the inflammatory pathogenesis that is potentially occurring within these intestines, as HAB toxins, such as microcystins, have been reported to cause severe gastrointestinal symptoms, even in humans. Exposure to HAB toxins such as microcystin in humans has led to gastroenteritis, abdominal pain, vomiting, and diarrhea [33,34]. Marked intestinal distension coupled with inflammatory processes and secretory diarrhea could account for the decreased amount of fecal contents within the intestines of HAB-toxin-exposed tadpoles. In fact, we have previously reported on the toxic effects of HAB toxins such as microcystins within the intestines, showing that microcystin actually exacerbates the inflammatory and toxic effects of inflammatory bowel disease, which supports our current findings [35].

One of the most notable pathologies observed within the livers of exposed tadpoles was hepatocyte hypertrophy. Hepatocyte hypertrophy can be a result of numerous pathological processes [36]. Interestingly, one of the pathologies that correlate with hepatocyte size is hepatocellular carcinoma [37], and hepatocellular hypertrophy is often the best single predictor of hepatocellular carcinoma in other experimental models [38]. In fact, HAB toxins such as microcystins have previously been identified as a liver tumor promoter [12]. Microcystins’ tumorigenic potential in the liver and previous correlations between hepatocyte size and hepatocellular carcinoma highlight the relevance of our finding of increased hepatocyte size within the livers of tadpoles exposed to HAB toxins.

Another liver finding seen in HAB-toxin-exposed tadpoles was an increase in hepatocyte binucleation. Binucleation or polyploidization can be a result of cellular stress from toxins, metabolic overload, or oxidative stress, and has been linked to many liver diseases [39]. It has been well established that one of the mechanisms of toxicity of HAB toxins such as microcystins is an increase in oxidative stress responses, which may account for the increased hepatocyte binucleation in the current study [40].

To further characterize the oxidative stress response to HAB toxins, we examined protein carbonylation in both the intestines and the livers of HAB-toxin-exposed tadpoles and found that it was elevated in both organs. Protein carbonylation, as measured by immunohistochemistry, is a hallmark of oxidative damage [41]. HAB toxins such as microcystins have been shown to act mechanistically to cause oxidative stress [40].

There have recently been several studies that have also investigated HAB toxin toxicity in amphibians, as this is a quickly growing area of concern. Junior et al. recently studied microcystin toxicity at a long-term exposure level of 16 days within *Lithobates catesbeiana* tadpoles [42]. Junior et al. reported that after 16 days of microcystin exposure, noticeable liver toxicity was observed. Consistent with our findings, Junior et al. observed hepatocellular hypertrophy and disruption of hepatocellular nuclei with fragmentation among other observations of necrosis, inflammatory cell infiltration, and fibrosis. In addition, Junior et al. also reported pathology within the gastrointestinal tract, including fibrosis, cellular apoptosis, and disruption of normal cellular architecture. These are important findings that show toxicity in the liver and gastrointestinal tract upon long-term exposure to microcystin. However, our findings are crucial in revealing that such toxicity in fact occurs with a much shorter exposure to HAB toxins containing microcystins. We report that just 7 days of exposure is sufficient in inducing toxicity within the liver and intestines, with several findings which support those reported by Junior et al., but our findings stress that hepatocellular and gastrointestinal damage actually begin much sooner than previously shown [42]. HAB toxin toxicity is largely regarded as a result of chronic exposure. However, our findings demonstrate that shorter exposures to HAB toxins containing microcystins can also begin causing toxic effects, highlighting the need to account for tadpoles and other species, potentially birds and other migratory animals, that do not necessarily grow and develop within HAB-toxin-contaminated waters but may ingest contaminated waters opportunistically.

Our findings are significant within the limited research currently published on HAB toxicity within amphibians, given that others have found HAB toxin, such as microcystins to have little to no effects in different amphibian species. Zikova et al. have previously investigated microcystins’ effects within *Xenopus laevis* (African clawed frog) tadpoles, a different species of tadpole [43]. They exposed tadpoles to microcystins for 1, 3, 7, and 21 days, which includes the 7-day exposure used in our study. None of the lengths of microcystin exposure were found to lead to changes in endocrine function, such as thyroid hormone regulation or sexual differentiation through Follicle Stimulating Hormone (FSH) and Luteinizing Hormone (LH) regulation. In addition, Zikova et al. reported only “minor to negligible” effects on development by the measurement of weight and developmental staging, or stress through the measurement of corticosterone and aldosterone levels. Along the same lines, Fischer et al. also studied the effects of microcystins in *Xenopus laevis* during their early life stages [44]. They reported that, with up to 5 days of microcystin exposure, no increases in mortality, malformation, or growth were observed. Zikova et al. and Fischer et al. observed little to no effects of microcystins on *Xenopus laevis* tadpoles and early-stage *Xenopus laevis*, which contrasts with the significant hepatic and gastrointestinal toxicity we observed in *Lithobates catesbeiana* tadpoles. Our results importantly highlight that, while short-term exposure to HAB toxins such as microcystins to some species has limited effects, other species, such as *Lithobates catesbeiana* tadpoles may be more susceptible and vulnerable to HAB toxicity [43].

It is important to note that HAB toxicity is a very real environmental hazard for tadpoles as it is indeed a significant part of their oral intake. Zhang et al. have previously investigated the oral intake of *Microcystis* by *Rana grylio* tadpoles in ponds within Guanqiao of Wuhan, China [45]. Within several eutrophic ponds within Guanqiao of Wuhan, Zhang et al. noticed significantly lower *Microcystis* levels in ponds with tadpoles than ponds without tadpoles. Within a laboratory environment, Zhang et al. confirmed that, when tadpoles were exposed to *Microcystis* suspensions, there was a 73% decrease in *Microcystis* levels in just 24 h. In addition, it is well known that the early stages of amphibian development occur in the shallow littoral zones of surface waters, and this is exactly where high levels or HAB toxins such as microcystins co-occur [44]. These confirm that tadpoles are a population and species that are directly exposed to and directly consume HAB toxins and are, therefore, truly at risk for HAB toxicity [45]. We also note that while we utilized 1 μg/L of total microcystin, the amount of total microcystins found in raw freshwater can range greatly depending on location, time of the year, and collection and detection methods. Concentrations range between detectable limits lower than the concentration used in this study to levels greatly exceeding this concentration, with reports up to 36 mg/L [1,10,46,47].

It is important to note some limitations within the current study. First, although we were able to accurately quantify the amount of extracellular, intracellular, and total microcystins, our study did not measure other potentially important parameters such as the total number of cyanobacterial cells present in the HAB material, chlorophyll content, or composition of minor species other than the dominant *Microcystis* cyanbacteria. Secondly, it is important to note that our HAB material exposure was adjusted based on total microcystin concentration (i.e., intracellular plus extracellular microcsytins) of the HAB material before addition to the terraria, and we did not measure dissolved microcsytins after addition of the HAB toxins to the terraria. Therefore, because a large percentage of the total microcystin was indeed intracellular (98.6%), the actual freely dissolved microcystin concentration to which the tadpoles were exposed may in fact be lower than the 1 μg/L total concentration stated. We also note that, while we and others have shown *Microcystis* to be the dominant species in our sample and, therefore, the focus of our investigation, there could also be potential effects from heterotrophic bacteria and cyanobacterial LPS. Though cyanobacterial LPS could contribute potential effects, previous studies have demonstrated that aquatic vertebrates such as amphibians and fish are resistant to LPS toxicity as many even lack a TLR4 ortholog and the costimulatory molecules for TLR4 activation by LPS [48]. Others have established that tadpoles are more resistant to LPS toxicity than adult frogs, showing that adult frog skin cells mount an inflammatory response to LPS through the production of interferon cytokines, whereas this response is absent in tadpole skin cells [49]. Wendel et al. conclude that tadpoles may be desensitized to the immune recognition and activation in response to LPS [49].

Collectively, our results show that HAB toxins, such as microcystins, have significant toxic effects within the intestines and livers of *Lithobates catesbeiana* tadpoles. While a recent report by Junior et al. showed that prolonged exposure of tadpoles (16 days) to microcystins induces toxic effects within the intestines and livers [42], our study and its findings demonstrate that toxicity by HAB toxins containing microcystins can be evident with an acute, short-term exposure (7 days) to HAB toxins. In contrast with other studies by Zikova et al. and Fischer et al., which did not find any microcystin toxicity in different species of tadpoles, our findings of significant toxicity highlight that some species, such as *Lithobates catesbeiana* tadpoles, may be more vulnerable and susceptible to the toxic effects of HAB toxins than others. These findings highlight the need to further investigate acute, short-term HAB toxin effects within populations at risk of exposure of HAB-toxin-contaminated waters and to be cognizant that some species may be more vulnerable and susceptible to HAB toxins’ effects than others.

## 4. Materials and Methods

### 4.1. HAB Toxin Collection and Characterization

Previous and concurrent studies have established that algal blooms in Lake Erie and the Maumee River are dominated by the genus Microcystis [50,51,52,53]. In particular, Chaffin et al. identified Microcystis from a massive HAB in the Western Basin of Lake Erie in 2011 using 200× magnification, used denaturing gradient gel electrophoresis to generate Microcystis-specific molecular fingerprints of the 16S-23S rRNA internal transcribed spacer region, and compared algal samples from across Lake Erie, the Maumee River, and sediment samples to identify the source population of Lake Erie’s annual algal blooms [54]. Their results demonstrate that the Microcystis populations throughout Lake Erie had high genetic similarity and likely originated from overwintering algal cells in the lake sediment; such overwintering cells act as an inoculum that fuels the subsequent year’s HAB. The National Oceanic and Atmospheric Administration (NOAA) tracks HAB events in Lake Erie using the Copernicus Sentinel-3 satellite and identifies the predominant species in a bloom based on species-specific thermal reflectance values calibrated by sampling algal cells and identifying them to species under a microscope. In 2017, the HAB in the Maumee River and Western Lake Erie was dominated by Microcystis, whereas the bloom near Sandusky Bay farther east was dominated by Planktothrix [55]

The HAB toxin material used for the current study was obtained in September 2017 from site L1 of the Maumee Bay area of Western Lake Erie (Lucas County, Ohio) as described in [50] during an unusually large HAB event in the Maumee River. Our sample was collected at the same location and within 2 days of the L1 sample analyzed by [50]. We used a plankton net to collect and concentrate algal scum from the upper water column at our sampling site. Description of the biomass and biovolume analysis of the algae at this site is provided in Palagama et al., and examination at 200× magnification confirmed the presence of Microcystis as the dominant genus in this HAB material. Furthermore, detailed characterization of the microcystin congeners contained in the HAB material used for this study using liquid chromatography–mass spectrometry and tandem mass spectrometry demonstrated that the most abundant microcystin congener in this material was MC-RR, followed by MC-LR [47,50].

Immediately after collection, we separated our algal sample into three aliquots and froze them. Total levels of total microcystins were established by passing the aliquot of unfiltered, concentrated algae through 3 freeze/thaw cycles in order to lyse cells, releasing all intracellular total microcystin. ELISA was then run on the sample, which accounts for the initially present, extracellular levels of total microcystin and the lysis-released, intracellular levels of total microcystin. The ELISA assay [55], which detects ADDA (3-amino-9-methoxy-2,6,8-trimethyl-10-phenyldeca-4,6-dienoic acid) levels, was performed on one aliquot to measure the combined intra- and extracellular total microcystin concentration following manufacturer instructions (Abraxis, Product No. 520011OH). This assay measured total microcystin concentration of 128 μg/L (intracellular was measured at 126.7 μg/L and extracellular was measured at 1.767 μg/L). Extracellular levels of total microcystins were quantified by filtering out all cells from the second aliquot of our algae sample using a 0.45 µm PVDF (polyvinylidene difluoride) filter. This sample was subsequently processed with the same 3 freeze/thaw cycles in order to assure equal processing. ELISA was then run to detect extracellular levels. Intracellular levels were determined by subtracting the extracellular levels from the total levels of total microcystin. The third aliquot was stored frozen to maintain toxicity until the tadpole exposure experiment was conducted in August 2018.

### 4.2. Tadpole Sampling and Treatment

All animals were captured and handled in accordance with Institutional Animal Care and Use Committee Protocol 108803 and Institutional Biosafety Committee Protocol 108801 (University of Toledo), Wild Animal Permit 21-016 (Ohio Department of Natural Resources), and Research Permit 2018010 (U.S. Fish and Wildlife Service), All animals were captured and handled in accordance with University of Toledo Institutional Animal Care and Use Committee Protocol 108803 (approved 6 March 2018), Institutional Biosafety Committee Protocol 108801, Wild Animal Permit 21-016 (Ohio Department of Natural Resources), and Research Permit 2018010 (U.S. Fish and Wildlife Service). Tadpole sampling and exposure were conducted as follows. Briefly, a total of 20 one-year-old American bullfrog (*Lithobates catesbeiana*) tadpoles were collected in August 2018 from Ottawa National Wildlife Refuge, Ottawa County, Ohio using a fyke net. Importantly, the collection pond had no direct contact or water exchange with Lake Erie and had not experienced harmful algal blooms. Therefore, the tadpoles were never exposed to HAB toxins. Once collected, the tadpoles were randomly divided into two experimental groups: control (n = 10) and HAB-toxin-exposed (n = 10). Tadpoles were housed in pairs in 5-gallon, plastic terraria filled with 2 gallons of pond water collected at the same time and locations as the tadpoles. We thawed the third aliquot of concentrated, unfiltered algae (collected as described above), re-froze and re-thawed it twice more for a total of 3 freeze–thaw cycles, and then, to each of the cages in the HAB-toxin-exposed group, we added 60 mL of concentrated, unfiltered algae to reach a final concentration of 1 μg/L of total microcystins in the terrarium water, the concentration at which detectable but sub-lethal effects have been observed in the congener *Rana nigromaculata* [56]. The 10 terraria of tadpoles (5 control and 5 HAB-toxin-exposed terraria) were maintained in a temperature-controlled room at 22 °C, on a 12-h light-dark cycle, in a secure Biosafety II room in the University of Toledo’s Department of Laboratory Animal Research Facility. Aquatic vegetation was added to each cage daily for food and cover. After seven days, the tadpoles were euthanized, and the intestines and livers from each tadpole were collected. Intestines and livers were immediately fixed in 10% neutral buffered formalin for 24 h and subsequently transferred to 70% ethanol.

As previous studies were limited and did not provide adequate estimates of effect size for the parameters measured in this species, after consultation with the University of Toledo’s IACUC we used the minimum number of animals that we reasonably expected would demonstrate statistical significance if our exposed group differed from the control group. As is standard practice for experiments on vertebrate animals from wild populations, the study was conducted only once to avoid negative impacts on the natural population.

### 4.3. Histology Preparation

The formalin-fixed intestinal and liver tissues were processed and embedded in paraffin (FFPE). Five (5)-micron tissue sections were placed on glass slides and stained with H&E. Images of histology slides were taken using an Olympus VS120 Slide Scanner.

### 4.4. Intestine Histopathological Analysis

Intestinal diameter, intestinal fold height, and intestinal fold number have been previously quantified in order to reflect gastrointestinal pathology [28]. Intestinal diameter quantification was completed using Olympus CellSens software (Standard 1.15) (Center Valley, PA, USA). Diameters were measured across the intestinal lumen from the apical membranes of intestinal epithelial cells from one side of the lumen to the opposing side. Ten (10) total cross-sectional diameter measurements were randomly taken from each tadpole intestine, and the average was obtained per tadpole.

The total number of intestinal folds per tadpole intestine was counted manually. The intestinal fold heights were measured from the outer edge of the intestinal wall to the peak of each fold using Olympus CellSens software. Twenty intestinal folds were measured randomly from each tadpole, intestine and averages were obtained per tadpole.

For fecal content density analysis, 5 representative images from the lumen of the gut of each tadpole were evaluated by FIJI biological image analysis software [57] (a distribution of ImageJ) [58] for their content density by thresholding on pixel brightness and were determined as Density (%) = ((Total image area − white space)/total image area) × 100.

### 4.5. Liver Histopathological Analysis

Hepatocyte size and hepatocyte bionucleation have been shown to reflect liver pathology [36,39]. Hepatocyte sizes were quantified using Olympus CellSens software. Hepatocyte areas were measured by delimiting the membranes of randomly selected hepatocytes. Fifty (50) hepatocytes were measured throughout each tadpole liver, and averages were obtained per tadpole.

The frequency of binucleated hepatocytes was quantified using Olympus CellSens software. Each liver was split into four quadrants. Square regions of about 600,000 µm^2^ were randomly drawn within each quadrant. The number of binucleated hepatocytes within each region were counted manually and the average number of binucleated hepatocytes per region was obtained for each tadpole liver.

### 4.6. Immunohistochemistry

Detection of protein oxidation by reactive oxygen species (ROS) was measured using the Protein Carbonyls Immunohistochemical Staining Kit (Cosmo Bio USA, Carlsbad, CA, USA, Catalog No. SML-ROIK04-EX). A goat, anti-rabbit IgG primary antibody (Vectastain Elite ABC HRP Immunodetection Kit, Novus Biologicals, LLC, Centennial, CO, USA, Catalog No. PK-6101-NB) was applied on each sample. Secondary solutions A and B (Vectastain Elite ABC HRP Immunodetection Kit) were subsequently applied. Slides were developed using HRP Color Development Reagent, DAB (Bio-Rad Laboratories, Hercules, CA, USA, Catalog No. 170-6535) and counterstained with hematoxylin (Fisher Scientific, Hampton, NH, USA, Catalog No. ICN10192225).

Photographs of whole tissue slides were taken at 20× magnification using a VS120 Virtual Slide Microscope (Olympus, Tokyo, Japan). Images were converted from .vsi to .tif and compressed using the Fiji distribution of ImageJ 1.52p (Wayne Rasband, National Institutes of Health, USA) [57]. Percent positive staining was determined as the number of positive shaded pixels (brown) divided by the total number of pixels representing the whole tissue multiplied by 100. All pixel analysis was performed using Image-Pro Analyzer 7.0 Software (Media Cybernetics, Rockville, MD, USA).

### 4.7. Statistical Analysis

Data presented are mean ± SD. Data obtained were first tested for normality via the Shapiro–Wilk and D’Agostino and Pearson normality tests. If the data did not pass the normality test, the Mann–Whitney Rank Sum test was used to compare the data. If the data did pass the normality test, parametric comparisons were performed using the unpaired Student’s *t*-test (two-tailed), as we have previously adhered to in previous investigations [59,60]. Equal variance was measured and confirmed in all data by F test. Statistical analysis was performed using GraphPad Prism 7.0d software (Graphpad Prism, San Diego, CA, USA). Significance was determined if *p* values were <0.05.
